# *Camelina sativa* L. Oil Mitigates Enteric *in vitro* Methane Production, Modulates Ruminal Fermentation, and Ruminal Bacterial Diversity in Buffaloes

**DOI:** 10.3389/fvets.2020.00550

**Published:** 2020-08-26

**Authors:** Hossam M. Ebeid, Faiz-ul Hassan, Mengwei Li, Lijuan Peng, Kaiping Peng, Xin Liang, Chengjian Yang

**Affiliations:** ^1^Key Laboratory of Buffalo Genetics, Breeding and Reproduction Technology, Ministry of Agriculture and Guangxi Buffalo Research Institute, Chinese Academy of Agricultural Sciences, Nanning, China; ^2^Dairy Science Department, National Research Centre, Giza, Egypt; ^3^Institute of Animal and Dairy Sciences, University of Agriculture, Faisalabad, Pakistan

**Keywords:** camelina oil, methane, rumen fermentation, methanogenesis, bacterial diversity

## Abstract

This study was aimed to evaluate the effects of *Camelina sativa* oil (CO) on fermentation kinetics and methane (CH_4_) production in rations with different roughage (R) to concentrate (C) ratios. Three total mixed rations (TMRs) were used as substrates (R70:C30, R50:C50, and R30:C70) supplemented with different levels of CO (0, 2, 4, 6, and 8% on dry matter basis) in an *in vitro* batch culture system. The enteric CH_4_ production was determined at different times of incubation while fermentation parameters were measured at the end of incubation. Results revealed that CO significantly decreased (*P* < 0.05) CH_4_ production at 48 h in medium (R50:C50) and low- (R30:C70) roughage diets than control. Camelina oil at all levels significantly (*P* < 0.05) affected ammonia nitrogen (NH_3_-N) and microbial protein (MCP) in all rations. Propionate concentration was increased by supplementing 8% CO to R70:C30 TMR, but it decreased with increasing levels of CO for low- and medium-roughage diets. Acetate concentration was significantly (*P* < 0.05) higher at 4% CO supplementation, but it decreased with 8% CO level in R30:C70 TMR. For all rations, CO decreased (*P* < 0.001) total bacteria, protozoa, and methanogens. Total fungi counts were affected by CO in all rations, especially with a 6% level in two rations (R30:C70 and R50:C50) and 8% level with high-roughage ration (R70:C30). Supplementation of CO in medium-roughage ration (R50:C50) showed a linear (*P* < 0.05) decrease in bacterial richness and evenness indices along with Shannon diversity as compared to the control. Moreover, CO also increased *Firmicutes* to *Bacteroidetes* ratio in all TMRs more effectively at higher levels. Camelina oil also affected the relative abundance of *Prevotella* in both low- and medium-roughage diets while increasing the abundance of *Ruminobacter* and *Pseudobutyrivibrio*. The present study concluded that CO enhanced fermentation kinetics while decreasing enteric *in vitro* CH_4_ production from fibrous diets. Thus, it may be considered as a potentially effective and environmentally friendly way of mitigating CH_4_ emission from livestock.

## Introduction

Rumen fermentation is a major contributor to enteric methane (CH_4_) in global greenhouse gas (GHG) emissions and considered an important player in the climate-change scenario. Methane mitigation has been keenly sought as a potential strategy to reduce GHG emissions while improving overall feed efficiency in ruminants. It is mainly targeted to mediate rumen biohydrogenation by diverting metabolic hydrogen away from methanogenesis toward the production of volatile fatty acids (VFA) that can potentially enhance production efficiency in ruminants and reduce environmental hazards. An earlier study has proposed that limiting methanogenesis can subsequently increase the production of microbial biomass (1). Moreover, excess metabolic hydrogen escaped from methanogenesis can be incorporated into NADH, which could be subsequently utilized in VFA synthesis and other fermentation end products in the rumen (2, 3).

Various methane mitigation strategies have been employed to control CH_4_ formation in the rumen including use of chemicals (4), plant extracts (5, 6), and supplementation of vegetable oils (7–9). Compared with other methane mitigation strategies, supplementation of dietary fat or vegetable oils is potentially advantageous in terms of increasing dietary energy in high-producing animals while limiting CH_4_ production (10, 11). Recently, the trend of incorporating fats as a source of dietary energy in place of carbohydrates is becoming popular, which also contributes to CH_4_ mitigation (12). The unsaturated fatty acid contents of plant seeds and/or their oils can desirably influence the biohydrogenation process in the rumen (13).

The effect of plant oils such as rapeseed, sunflower, soybean, and linseed oils on rumen fermentation in diets with different roughage: concentrate ratios (R:C ratio) has been extensively studied (14–17). Studies have revealed that oilseeds can be one of the efficient ways to reduce enteric CH_4_ production from ruminants as they can mitigate CH_4_ by directly inhibiting rumen protozoa and methanogens while increasing biohydrogenation of polyunsaturated fatty acids (PUFA) to act as a sink for hydrogen produced by rumen microbes (8, 16, 18). The oil of *Camelina sativa* L. seed (CS), also known as false flax seed, is a rich source of unsaturated fatty acids (68–74%) especially oleic, linoleic, and linolenic acids (19, 20). This rich unsaturated fatty acid profile makes CS a high-quality fat supplement for ruminants. Moreover, CO is a quite stable oil despite having high PUFA contents owing to its rich antioxidant profile (21). Several studies have reported the use of CS meal or cake as a protein source in beef, lactating sheep, and dairy cow rations (22–24) while few have also used whole seed as a feed supplement (14, 20).

Studies have reported the nutritional benefits of CS on animal performance, but information regarding its potential effects on fermentation kinetics and ruminal microbiota is limited (13). Few studies have investigated the effect of oil extracted from whole camelina seed in dairy cows fed grass silage (25) and lambs (15). No study is available on the effect of CO on methane mitigation in high fibrous diets.

We hypothesized that PUFA contents (linolenic acid) of CO could potentially reduce CH_4_ production by mediating rumen biohydrogenation and microflora. Therefore, we aimed to evaluate the effect of camelina oil on *in vitro* CH_4_ production, rumen fermentation, and microbial populations in total mix rations (TMRs) with different roughage-to-concentrate ratios.

## Materials and Methods

### Substrates and Experimental Design

Three total mixed rations were prepared with different roughage-to-concentrate ratios, viz., 70:30, 50:50, and 30:70 coded as R70:C30, R50:C50, and R30:C70, respectively. Details of experimental TMRs are given in Table 1.

**Table 1:** Ingredients and chemical composition (g/kg DM) of the three total mixed rations with different elephant (R) to concentrate (C) ratios (*N* = 3).

**Items**	**Ingredient**		**Ration (R:C)**		
	**Elephant grass**	**CFM^**a**^**	**70:30**	**50:50**	**30:70**
**Ingredient**					
Elephant grass			700	500	300
Crushed corn			180	300	420
Wheat bran			33	55	77
Soybean meal			75	125	175
Shell powder			1.5	2.5	3.5
Dicalcium phosphate			4.5	7.5	10.5
Sodium chloride			3	5	7
Minerals/vitamins mixture^b^			3	5	7
**Chemical composition**					
Dry matter	966	947	960	956	952
Organic matter	872	789	847	830	814
Crude protein	63	163	93	113	133
Neutral detergent fiber	879	595	794	737	680
Acid detergent fiber	532	126	410	329	248
GE^c^ (kcal/kg DM)	4.16	3.95	4.06	4.01	4.06

a *CFM, Concentrate feed mixture*.

b *Contained per kg: vitamin A 550 000 IU, vitamin E 3000 IU, vitamin D3 150 000IU, 4.0 g Fe (as ferrous sulfate), 1.3 g Cu (as copper sulfate), 3.0 g Mn (as manganese sulfate), 6.0 g Zn (as zinc sulfate), 80 mg Co (as cobalt sulfate)*.

c *GE, gross energy*.

Camelina oil was obtained from Mountain Rose Herbs Company, Denmark. An emulsion (oil-in-water) of CO was prepared using the ultrasonic bath (Sonics Vibra-Cell™, USA) by suspending in distilled water (1:9; v/v). This emulsion was supplemented in substrates at four levels including 2% (125 μL), 4% (250 μL), 6% (375 μL), and 8% (500 μL) on dry matter (DM) basis. Two *in vitro* batch culture runs were carried out separately for each ration with oil supplement. Each oil level was tested in three replicates with three blank vessels (no substrate) for each run (in total 6 replicates). Five-hundred milligram of each ration was taken in the bottle (180 mL) as a substrate, and an artificial buffer solution was added, followed by mixing ruminal fluid at a 4:1 ratio as reported previously (26). The buffer solution was prepared 1 day before starting the fermentation. The rumen fluid collected from three cannulated water buffaloes (Murrah × Chinese local with an average body weight of 450 kg) fed the same roughage and concentrate diet was used for *in vitro* fermentation. The ruminal digesta was collected from different places of the rumen in clean containers and strained through four layers of cheesecloth to get rumen fluid while flushing with CO_2_. A 50-mL ruminal buffer solution was injected into each bottle with flushing CO_2_, and after sealing, the bottles were kept in a pre-warmed incubator (at 39°C) for the next 48 h.

### Sample Collection

During the incubation period, a 10-μL gas sample was taken from each bottle with a gas-tight syringe and manually injected into the GC system to determine the CH_4_ concentration at 3, 6, 9, 12, 24, and 48 h of incubation using the GC system (7890A, Agilent Technologies, USA) with a capillary column measuring 30 m × 0.32 × 0.25 mm film thickness (GC-14B, Shimadzu, USA). Five different concentrations of pure CH_4_ gas standards were used to develop calibration curves for the calculation of total CH_4_ produced. The total gas production volume was measured by a glass syringe (100 mL) before each CH_4_ measurement (data of gas production not shown).

After 48 h of incubation, the pH of rumen fluid was measured by a pH meter (HI 9024C; HANNA Instruments, Woonsocket, Rhode Island, USA), immediately after the opening of bottles. Samples of rumen fluid were separated into labeled plastic tubes (15 mL) for the analyses of microbial crude protein (MCP), ammonia nitrogen (NH_3_-N), and volatile fatty acid fractions including acetic (C2), propionic (C3), butyric (C4), and valeric (C5) acids and their isomers including isobutyric (iC4) and isovaleric (iC5) acids. After that, all the samples were stored at −20°C until further processing.

### Chemical Analyses

The fatty acid profile of camelina seed oil samples was analyzed as described previously (27) and presented in Table 2. Briefly, 1.0 mL of n-hexane was added to 15 mg of CO and vortexed for 30 s followed by the addition of 1 mL of sodium methoxide (0.4 mol). The mixture was allowed to settle for 15 min after vortexing for 30 s. The upper phase, containing the fatty acid methyl ester (FAME), was recovered and analyzed by an Agilent 7890B gas chromatography (GC-FID) with a polar capillary column SP®-2560 100 m, 0.25 mm id, 0.2 μm film thickness. Helium was used as a carrier gas at a flow rate of 20 cm s-1 and split ratio 100:1. The column temperature profile was held at 100°C for 5 min, ramp to 240°C at 4°C min-1, and held at 240°C for 30 min. A sample volume of 1.0 μL was injected. The FAME was identified by comparing their relative and absolute retention times with FAME standards (from C4:0 to C24:0).

**Table 2:** Fatty acid profile of oil isolated from *camelina sativa* (L) seeds (mean ± SD) (*N* = *3*).

**Fatty acid**	**Concentration (g/kg^**a**^)**
Palmitotate acid	70.9 ± 3.49
Stearic acid	28.1 ± 7.28
Oleic acid	168.7 ± 9.54
Linoleic acid	178.1 ± 11.6
Linolenic acid	302.5 ± 1.45
Arachidic acid	26.1 ± 2.02
Arachidonic acid	139.5 ± 5.23
Eicosadienoic acid	25.4 ± 3.62
Eicosatrienoic acid	16.8 ± 0.29
Behenic acid	6.2 ± 2.14
Saturated fatty acids	131.3 ± 14.93
Unsaturated fatty acids	831.0 ± 31.73
Other	37.7 ± 5.62

a *Concentration based on the total areas of the identified peak*.

The proximate analysis of TMRs was carried out for DM (ID: 934.01), ash (ID: 942.05), and nitrogen (ID: 954.01), and ether extract (ID: 920.39) according to AOAC procedures (28). The TMR samples were also analyzed for neutral detergent fiber (NDF) and acid detergent fiber (ADF) (ID: 973.18), as described by Van Soest et al. (29) using an ANKOM^2000^ Fiber Analyzer Unit (ANKOM Technology Corp., Macedon, NY, USA). Neutral detergent fiber content was analyzed with heat-stable α-amylase and sodium sulfite per sample in the neutral detergent solution. The NDF and ADF were expressed, inclusive of residual ash. The gross energy (GE) of rations was determined by a bomb calorimeter (PARR Calorimeter, USA).

Samples of VFA fractions in rumen fluid (C2, C3, C4, C5, iC4, and iC5) were measured using the GC system (Model 7890A, Agilent Technologies, USA; column temperature = 120°C, injector temperature = 180°C, detector temperature = 180°C). 0.5 mL of meta-phosphoric acid (25%) was added to 1 mL of filtrate then centrifuged (1,2000 × g for 10 min), and supernatant (920 μL) plus 80 μL crotonic acid (as internal standard) in a GC bottle for a volatile fatty acid determination as reported previously (30). A portion of 4 mL filtrate was acidified with 4 mL of HCl (0.2 mol/L) and stored in a freezer (−20°C) for NH_3_-N using the indophenol method (31). For the determination of MCP, a 5-mL filtrate was then centrifuged at 800–1,000 rpm for 5 min at 4°C to remove feed particles. Then, 1.5 ml of supernatant was centrifuged at 12,000 rpm (4°C) for 15 min to collect the microbial biomass. After that, 0.5 mL (0.25 N) NaOH was added to the microbial biomass, mixed, and heated at 100°C in a water bath for 20 min. After this treatment, the mixture was centrifuged at 12,000 rpm (4°C) for 30 min, and the supernatant was collected for microbial CP analysis using the colorimetric method. Briefly, 100 μL supernatant was added to 5 mL Coomassie Brilliant Blue (G250, 95% ethanol, 85% phosphoric acid, and double-distilled water) and mixed well. Absorbance at 595 nm was checked by a 721 spectrophotometer colorimeter using 1 mg/ml bovine serum albumin solution (Sigma-Aldrich Co., LLC, St. Louis, Missouri, USA) as a standard equivalent (32). DNA of ruminal microbes was extracted from 1 mL of frozen samples of batch culture filtrate using the CTAB bead beating method (33).

### Determination of Microbial Populations Using Real-Time PCR

Microbial populations in batch culture filtrate were determined through quantitative real-time PCR (qRT-PCR) by using a Roche LightCycler 480 RT-PCR machine (Roche, Basel, Switzerland). For the determination of methanogens and bacteria, we used a previously reported 16S-rRNA primer while for anaerobic fungi and protozoa, 18S-rRNA primers were used (34–36). Details of primers used in our study are presented in Table 3, while remaining procedures, including RT-PCR amplification profile and reaction mixture, were performed as reported in our previous study (8).

**Table 3:** PCR primers for real-time PCR assay.

**Target strain**	**^**a**^Pre/post primer**	**Primer sequence**	**Amplification length (bp)**	**References**
Total bacteria	F	CGGCAACGACCGCAACCC	130	(35)
	R	CCATTGTAGCACGTGTGTAGCC		
Total fungi	F	GAGGAAGTAAAAGTCGTAACAAGGTTTC	120	(35)
	R	CAAATTCACAAAGGGTAGGATGATT		
Total protozoa	F	GCTTTCGWTGGTAGTGTATT	223	(34)
	R	CTTGCCCTCYAATCGTWCT		
Total methanogens	F	TTCGGTGGATCDCARAGRGC	140	(36)
	R	GBARGTCGWAWCCGTAGAATCC		

a *F, forward; R, reverse*.

### Sequencing of 16S-rRNA Gene for Determination of Rumen Bacterial Diversity

The DNA samples from one lower level (2%) and one higher level (8%) of CO were used for 16S rRNA gene sequencing to determine bacterial diversity. High-throughput (Illumina MiSeq) sequencing of the 16S rRNA gene was carried out using barcoded primers for the V3–V4 region (37). DNA libraries were sequenced using a 2 × 300 paired-end sequencing module (Illumina, San Diego). The taxonomic assignment of cleaned sequences was performed by aligning them against the SILVA database (Release128) using the Ribosomal Database Project (RDP) Classifier (http://rdp.cme.msu.edu/). Data about operational taxonomic units (OTU) were grouped taxonomically (at phylum and genus) for all treatment groups. The taxonomic classification of rumen bacteria in the steps mentioned above was performed as previously reported (38) using Qiime software (http://qiime.org/scripts/assign_taxonomy.htmL). Rarefaction curves and community bar plots were generated using R software (v2.3.2). Moreover, MOTHUR (V 1.31.2) software was used to analyze alpha diversity parameters.

### Statistical Analysis

For each TMR, values recorded from three replicates of incubation run were averaged. Thus, within each TMR, there were six replicates per oil level (each corresponding to the average value recorded at two incubation runs), and each replicate was considered as an experimental unit. The effect of treatment on *in vitro* CH_4_, rumen fermentation, and alpha bacterial diversity parameters of each TMR was analyzed through the PROC GLM procedure of SAS (SAS Institute Inc., Cary, NC, USA, version 14, 2015) using the following mixed model:

(1) $$\begin{array}{l} \text{Yijk}=\mu +\text{TMRi}+\text{COj}+\text{Eijk} \end{array}$$

where Yijk = is every observation of the *i*th TMR type (TMRi) with *j*th CO level (COj); μ is the overall mean; Eijk is the experimental error. Copy numbers of protozoa, methanogens, bacteria, and fungi were log-transformed, and then a Poisson regression model was fitted using the PROC GENMOD of SAS. The level of statistical significance for all analyses was *P* < 0.05.

## Results

### Chemical Composition of Diets and Fatty Acid Profile of Camelina Oil

Increasing the level of roughage ratio (elephant grass) in TMR decreased crude protein and increased NDF and ADF contents (Table 1). However, DM, OM, and GE were similar among the three TMRs. Analysis of fatty acid profile revealed linolenic acid as the most abundant fatty acid followed by linoleic, oleic, and arachidonic acids in CO (Table 2). Total unsaturated fatty acid (UFA) concentrations of CO were far greater than the total saturated fatty acids (831 vs. 131 mg/kg).

### *In vitro* Methane Production

Treatment significantly affected the *in vitro* CH_4_ production in three rations at different hours of incubation (Table 4). For ration R70:C30, CO linearly increased (*P* < 0.05) CH_4_ production at 3, 6, 9, and 12 h of incubation than control. However, after 48 h of incubation CH_4_ production was linearly (*P* > 0.05) decreased with supplementation of CO (Table 4). For ration R50:C50, CO almost linearly (*P* < 0.001) decreased CH_4_ production after 24 h of incubation than control. For ration R30:C70, CO linearly increased (*P* < 0.05) CH_4_ production at 3, 6, 9, and 12 h of incubation but linearly (*P* < 0.001) decreased CH_4_ production after 48 h of incubation than control. Moreover, all TMRs exhibited a decrease in CH_4_ production with all CO levels compared to the control after 48 h.

**Table 4:** *In vitro* methane (CH_4_) production (mL) kinetics of three total mixed rations supplemented with different levels of camelina oil (*N* = 6).

**Time/h**	**Oil levels**					**SEM**	***P*-value**
	**0%**	**2%**	**4%**	**6%**	**8%**		
**Roughage:concentrate (70:30)**							
3	9.73b	9.99b	10.38a	9.91b	10.46a	0.075	0.002
6	10.53c	11.25ab	11.15b	11.22ab	11.64a	0.094	0.001
9	12.39c	13.71ab	14.23a	13.45b	13.80ab	0.155	0.001
12	14.78b	15.14ab	15.40a	15.30ab	15.35a	0.084	0.130
24	21.23	22.16	22.34	21.95	22.33	0.187	0.312
48	28.72	27.31	28.41	27.70	26.74	0.406	0.566
**Roughage:concentrate (50:50)**							
3	9.39	9.76	9.59	9.78	9.77	0.056	0.116
6	11.60	11.44	11.61	11.92	11.78	0.075	0.312
9	13.35	13.98	14.15	14.48	14.46	0.103	0.196
12	16.99	16.61	17.17	16.68	16.85	0.193	0.905
24	24.04a	23.09b	22.76b	23.16b	22.52b	0.137	0.001
48	26.45a	25.60b	25.48bc	25.63b	24.86c	0.130	0.001
**Roughage:concentrate (30:70)**							
3	9.21c	9.51b	9.47bc	9.44bc	9.81a	0.053	0.003
6	10.68c	10.92bc	10.98abc	11.23ab	11.25a	0.059	0.005
9	12.93b	13.13b	13.40ab	13.84a	13.47ab	0.097	0.024
12	15.43c	15.66bc	15.74bc	15.52a	16.17ab	0.111	0.007
24	24.78	24.66	24.54	24.91	24.53	0.127	0.876
48	30.78a	29.89ab	30.30ab	29.59b	29.18b	0.198	0.058

*Arithmetic mean in the same row within each ration with different letters differ significantly (P < 0.05)*.

### *In vitro* Rumen Fermentation Kinetics

Ruminal pH did not differ among treatments with both high- and medium-roughage rations, but increasing levels of CO linearly (*P* < 0.0001) decreased the pH in low-roughage ration (Table 5). Dietary CO levels, as well as the type of ration, significantly affected NH_3_-N and MCP concentrations (Table 5). The concentrations of NH_3_-N and MCP were increased with the supplementation of CO in low- and medium-roughage rations. On the other hand, both NH_3_-N and MCP concentrations were linearly decreased with CO supplementation in the high-roughage ration. The concentration of total VFAs increased with the increase in concentrate ratio in TMRs. Dietary supplementation of CO at 4% in high-roughage ration showed a significant increase in total VFAs. For ration R70:C30, propionic acid was linearly increased (*P* < 0.016) with an increasing level of CO; however, the A/P ratio was decreased (*P* < 0.05) with 8% CO as compared to the control. For the R50:C50 ration, 2% CO increased (*p* < 0.018) the concentration of C3, while higher levels showed a negative effect on C3 yield. The C4 (*P* < 0.003), C5 branched-chain VFA (*P* < 0.031), and C5 linearly (*P* < 0.001) decreased with the increasing level of CO. For R30:C70 TMR, 4% of CO significantly increased the cross C2, C4, and C5 branched-chain VFA and C5, while significantly decreasing (*P* < 0.014) the C3 concentration.

**Table 5:** *In vitro* fermentation parameters of three total mixed rations supplemented with different levels of camelina oil (*N* = 6).

Items^*^	**Oil levels**					**SEM**	***P*-value**
	**0%**	**2%**	**4%**	**6%**	**8%**		
**Roughage: concentrate (70:30)**							
pH	6.75	6.75	6.74	6.70	6.74	0.010	0.644
NH_3_-N	7.93a	7.92a	7.80a	6.25b	6.69ab	0.227	0.031
MCP	12.08a	11.89a	10.87ab	10.87ab	9.24b	0.351	0.067
TVFA	24.24	22.22	23.87	25.19	24.90	0.467	0.299
C2	11.16	10.11	10.87	11.53	11.29	0.221	0.312
C3	7.94ab	7.40b	7.89ab	8.43ab	8.48a	0.155	0.016
isoC4	0.47	0.44	0.47	0.47	0.46	0.007	0.609
C4	3.99	3.66	3.96	4.11	4.02	0.079	0.462
isoC5	0.41	0.37	0.42	0.40	0.40	0.011	0.705
C5	0.26	0.22	0.25	0.25	0.26	0.006	0.596
A/P	1.40a	1.36ab	1.37ab	1.37ab	1.33b	0.008	0.050
**Roughage: concentrate (50:50)**							
pH	6.75	6.76	6.75	6.76	6.74	0.003	0.134
NH_3_-N	8.84a	7.77b	7.43b	8.92a	9.29a	0.202	0.005
MCP	9.20ab	8.57b	8.70b	9.57ab	10.79a	0.290	0.099
TVFA	26.23	26.27	25.32	26.02	25.59	0.157	0.230
C2	11.66	11.49	11.17	11.42	11.19	0.078	0.229
C3	8.67ab	8.96a	8.32b	8.63ab	8.58ab	0.082	0.018
isoC4	0.50a	0.49ab	0.47c	0.47bc	0.46c	0.004	0.003
C4	4.51	4.48	4.59	4.71	4.60	0.042	0.478
isoC5	0.51a	0.49ab	0.45b	0.46b	0.45b	0.008	0.031
C5	0.37a	0.35ab	0.32c	0.34bc	0.31c	0.006	0.001
A/P	1.35	1.28	1.35	1.33	1.31	0.011	0.293
**Roughage: concentrate (30:70)**							
pH	6.68ab	6.70a	6.66bc	6.64d	6.62dc	0.007	<0.0001
NH_3_-N	9.16a	6.31b	9.48a	9.33a	11.07a	0.414	0.0021
MCP	10.18	9.89	9.15	10.33	10.12	0.257	0.6479
TVFA	30.06a	28.95ab	30.24a	29.69a	27.88b	0.253	0.0099
C2	12.82a	12.40ab	12.91a	12.65a	11.87b	0.112	0.0132
C3	10.31a	9.75ab	10.21a	10.06a	9.32b	0.108	0.0144
isoC4	0.543a	0.543a	0.558a	0.533ab	0.507b	0.006	0.0504
C4	5.33	5.13	5.38	5.38	5.16	0.047	0.2749
isoC5	0.647ab	0.680a	0.687a	0.645ab	0.607b	0.011	0.1263
C5	0.420b	0.453ab	0.495a	0.427b	0.417b	0.010	0.0392
A/P	1.24	1.27	1.27	1.26	1.28	0.008	0.7449

*Arithmetic mean in the same column within each ration with different letters differ significantly (P < 0.05)*.

* *Items: NH_3_-N, ammonia-N (mg /100 mL); MCP, microbial crud protein (mg/mL); VFA, volatile fatty acids (mmol/L); C2, acetic acid (mmol/L); C3, propionic acid (mmol/L); C4, butyric acid (mmol/L); C5, valeric acid (mmol/L); iC4, isobutyric acid (mmol/L); iC5, isovaleric acid (mmol/L); A/P, acetate to propionate ratio*.

### *In vitro* Rumen Microbial Populations

According to qRT-PCR results, type of ration and the level of CO significantly (linear and quadratic effects, *P* < 0.05) affected rumen microbial populations (Table 6). The number of protozoa, methanogens, and total bacteria was linearly and quadratically decreased (*P* < 0.0001) with CO supplementation in all rations. However, 8% CO increased the number of protozoa, methanogens, and total bacteria as compared to the control. Moreover, CO affected the total fungal counts in all rations, especially at 6% in the low-roughage ration (R30:C70) and 8% level in the high-roughage ration (R70:C30).

**Table 6:** Ruminal microbiota population (log colony forming units/mL) of three total mixed rations supplemented with different levels of camelina oil (*N* = 3).

**Ration**	**Level**	**Protozoa**	**Methanogens**	**Bacteria**	**Fungi**
**R70:C30**	0	8.21a	7.08a	20.37a	5.11ab
	2	7.49b	6.51b	20.24b	5.16a
	4	7.03c	6.67b	18.31e	5.17a
	6	7.47b	6.67b	19.81c	5.13ab
	8	6.79d	6.32c	19.05d	5.03b
	SEM	0.017	0.022	0.0004	0.044
	Oil linear	<0.0001	<0.0001	<0.0001	0.225
	Oil quadratic	<0.0001	0.001	<0.0001	0.032
**R50:C50**	0	8.63a	6.95a	21.15a	5.19
	2	8.50b	6.27d	19.81e	5.11
	4	8.16d	6.60c	20.18c	5.12
	6	8.31c	6.75b	20.74b	5.07
	8	7.89e	6.28d	20.08d	5.11
	SEM	0.009	0.017	0.0003	0.043
	Oil linear	<0.0001	<0.0001	<0.0001	0.132
	Oil quadratic	<0.0001	0.003	<0.0001	0.281
**R30:C70**	0	7.51b	6.54c	18.49b	5.18a
	2	7.45c	7.04a	17.95e	5.24a
	4	7.07d	5.98d	17.96d	5.20a
	6	7.02d	6.59c	17.98c	5.02b
	8	7.77a	6.73b	19.49a	5.13ab
	SEM	0.016	0.021	0.0001	0.043
	Oil linear	0.017	0.375	<0.0001	0.016
	Oil quadratic	<0.0001	<0.0001	<0.0001	0.865
***P*****-value Ration**					
	SEM	0.005	0.001	0.0003	0.002
	Linear	0.0003	<0.0001	<0.0001	<0.0001
	Quadratic	<0.0001	<0.0001	<0.0001	<0.0001
**Oil effect**					
	SEM	0.006	0.001	0.0003	0.002
	Linear	<0.0001	<0.0001	<0.0001	<0.0001
	Quadratic	<0.0001	<0.0001	<0.0001	<0.0001

*Arithmetic mean in the same column within each ration with different letters differ significantly (P < 0.05)*.

### *In vitro* Bacterial Diversity

#### Taxonomy Statistics and Rarefaction Curves

**Figure 1:**
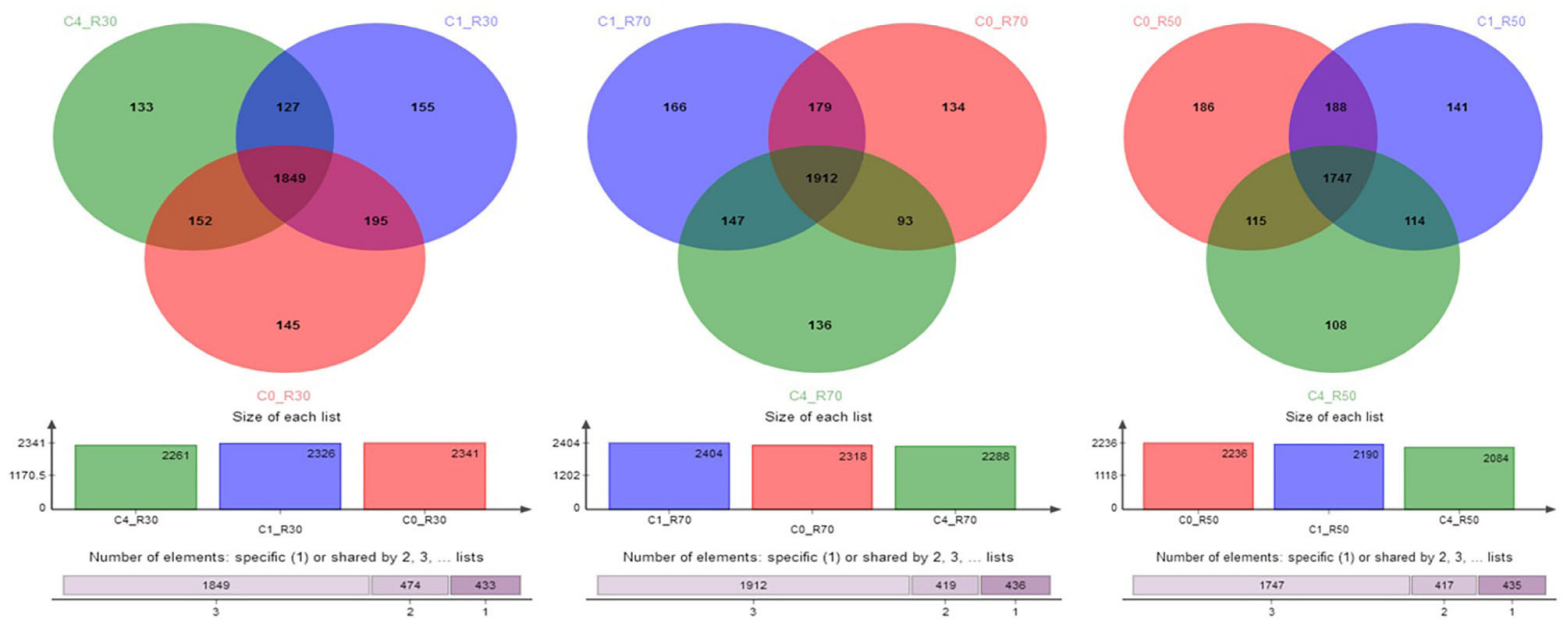
Shared and unique OTUs across groups. C0_R70: control with 70% high roughage; C1_R70: 1% camelina oil level with 70% high roughage; C4_R70: 4% camelina oil level with 70% high roughage; C0_R30: control with 30% low roughage; C1_R30: M1: 1% camelina oil level with 30% low roughage; C4_R30: 4% camelina oil level with 30% low roughage; C0_R50: control with 50% medium roughage; C1_R50: M1: 1% camelina oil level with 50% medium roughage; C4_R50: 4% camelina oil level with 50% medium roughage.

**Figure 2:**
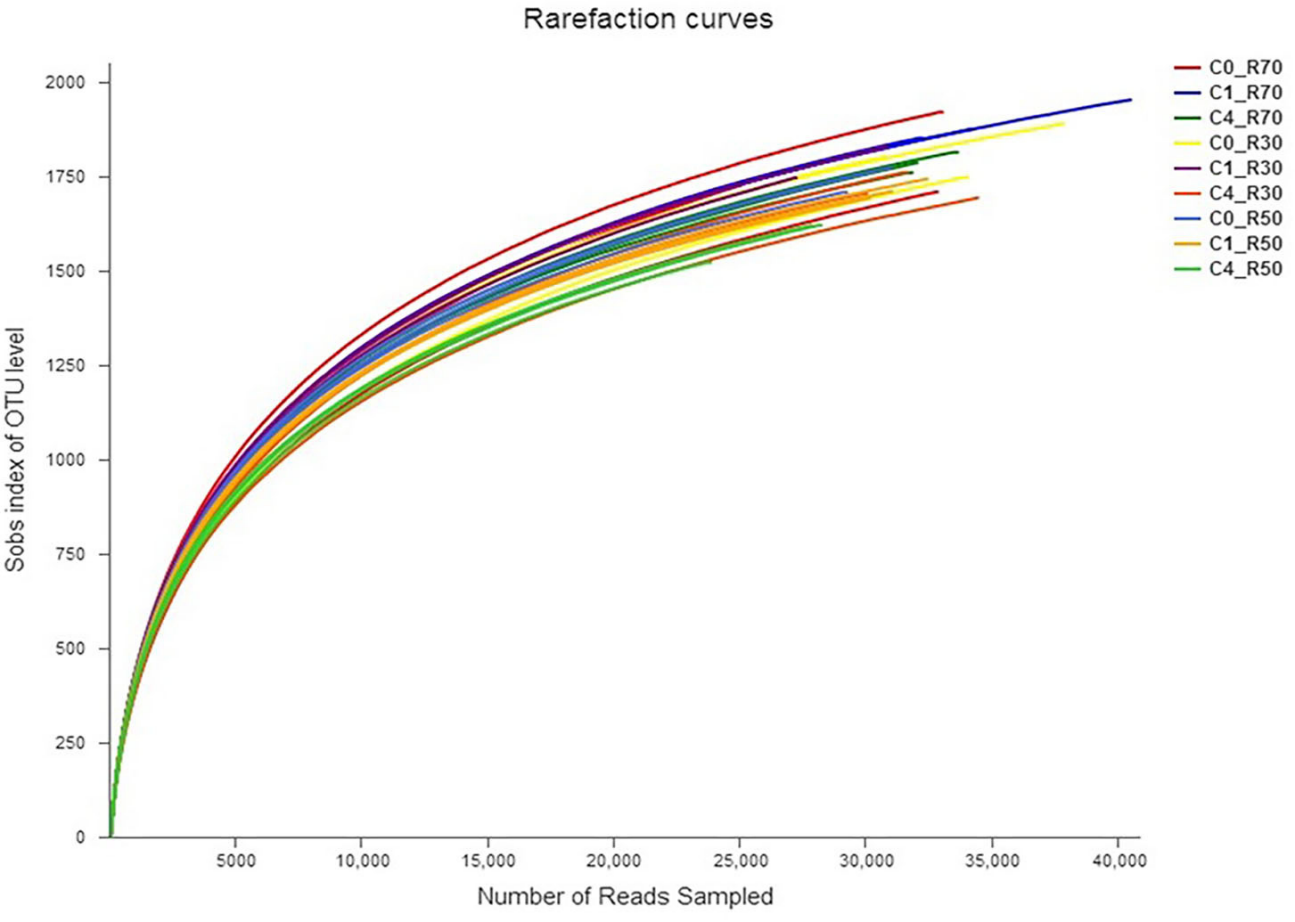
Rarefaction curve showing bacterial richness across samples. C0_R70: control with 70% high roughage; C1_R70: 1% camelina oil level with 70% high roughage; C4_R70: 4% camelina oil level with 70% high roughage; C0_R30: control with 30% low roughage; C1_R30: M1: 1% camelina oil level with 30% low roughage; C4_R30: 4% camelina oil level with 30% low roughage; C0_R50: control with 50% medium roughage; C1_R50: M1: 1% camelina oil level with 50% medium roughage; C4_R50: 4% camelina oil level with 50% medium roughage.

A total of 3164 OTU were identified through analysis of 16S-rRNA gene sequence data in the three TMRs supplemented with three CO levels (0, 2%, and 8%). The taxonomic data revealed 23 phyla, 40 classes, 86 orders, 157 families, 364 genera, and 719 species of rumen bacteria detected in rumen filtrate. Shared and unique OTU for three rations with different levels of CO are presented in Figure 1. The total number of OTU increased in the high-roughage ration (R70) in both control and supplemented groups. The higher number of unique OTUs individually identified in the control group was observed with a medium-roughage ration followed by low CO level with high- and low-roughage rations, respectively. The number of observed species (sobs) at the OTU level was greater in the high-roughage ration (R70:C30) as compared to other groups (Figure 2).

#### Bacterial Diversity Indices

Results of alpha bacterial diversity indices are presented in Table 7. For the R70:C30 diet, the richness (sobs, ace, and Chao) and diversity (Shannon and Simpson) indices were increased (*P* > 0.05) with supplementation of the lower level of CO (2%) as compared to the high level (8%) and control in a high-roughage diet (R70). On the other hand, the treatment showed a linear (*P* < 0.05) decrease in all richness and evenness indices as well as Shannon diversity in the medium roughage ration (R50:C50). However, the low-roughage ration (R30:C70) showed a similar trend for richness and Shannon indices that were observed in the high-roughage diet (R70) supplemented with a 2% CO level as compared to others. The ace index was significantly (*P* < 0.042) increased with a 2% CO level.

**Table 7:** Effect of camelina oil supplementation and three total mixed rations alpha microbial diversity estimators (*N* = 3).

Level^*^	**sobs**	**Shannon**	**Simpson**	**ace**	**Chao**	**coverage**	**Shannon eveness**
**Roughage: concentrate (70:30)**							
C	1785.00	6.02	0.0076	2248.72	2292.80	0.983	0.804
L	1893.33	6.06	0.0078	2273.03	2312.65	0.986	0.804
H	1786.66	6.05	0.0078	2176.16	2219.93	0.986	0.808
SEM	43.80	0.056	0.0006	38.27	46.15	0.001	0.005
Linear	0.615	0.797	0.833	0.150	0.226	0.331	0.596
Quadratic	0.101	0.603	0.843	0.417	0.541	0.134	0.889
**Roughage: concentrate (50:50)**							
C	1748.00a	6.17a	0.005	2148.78a	2156.79	0.985a	0.826a
L	1715.33a	6.11a	0.005	2079.76ab	2104.00	0.986a	0.821a
H	1587.33b	6.01b	0.006	1980.15b	2002.11	0.983b	0.815b
SEM	23.79	0.017	0.0003	31.41	43.00	0.0004	0.002
Linear	0.002	0.001	0.034	0.009	0.042	0.022	0.010
Quadratic	0.813	0.536	0.493	0.527	0.805	0.023	0.362
**Roughage: concentrate (30:70)**							
C	1813.33	6.127	0.005	2205.30a	2214.71	0.987	0.816
L	1800.66	6.198	0.004	2238.12a	2276.18	0.984	0.826
H	1719.33	5.99	0.007	2110.98b	2154.61	0.986	0.804
SEM	31.01	0.052	0.001	27.91	41.67	0.001	0.006
Linear	0.060	0.056	0.039	0.025	0.190	0.920	0.091
Quadratic	0.793	0.168	0.193	0.164	0.199	0.026	0.134
***P*****-value Ration**							
SEM	19.57	0.02	0.0003	18.94	25.20	0.0005	0.002
Linear	0.130	0.116	0.0003	0.091	0.109	0.930	0.017
Quadratic	0.0001	0.477	0.041	<0.0001	<0.0001	0.637	0.008
**Camelina oil**							
SEM	19.57	0.02	0.0003	18.94	25.20	0.0005	0.002
Linear	0.002	0.011	0.012	0.0002	0.006	0.882	0.077
Quadratic	0.108	0.214	0.668	0.333	0.311	0.440	0.393

*Arithmetic mean in the same column within each ration with different letters differ significantly (P < 0.05)*.

* *Level: C, control without CO; L, low, 2% CO; H, high, 8% CO level*.

#### The Relative Abundance of Bacterial Phyla

**Figure 3:**
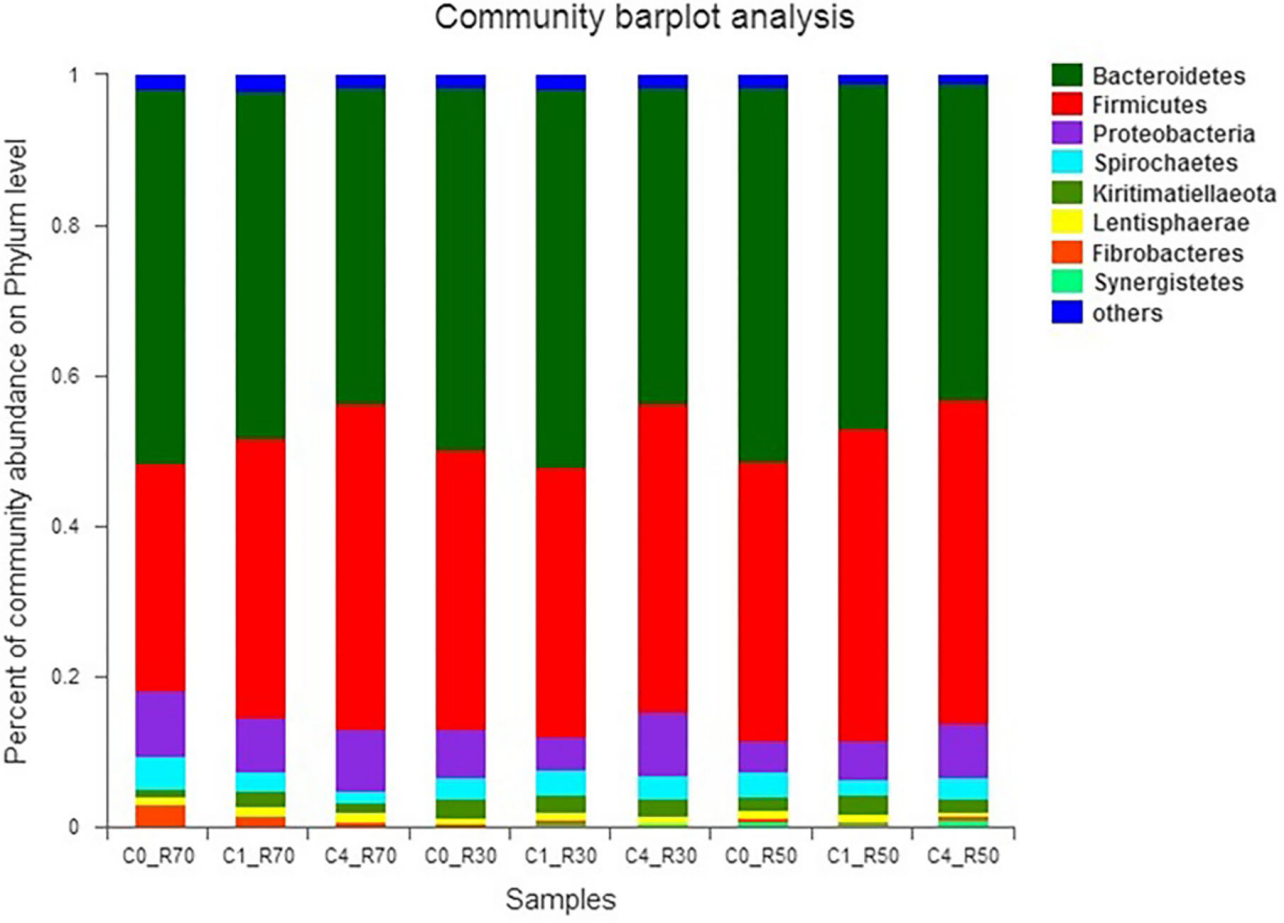
Relative abundance of bacterial phyla across different treatment groups. C0_R70: control with 70% high roughage; C1_R70: 1% camelina oil level with 70% high roughage; C4_R70: 4% camelina oil level with 70% high roughage; C0_R30: control with 30% low roughage; C1_R30: M1: 1% camelina oil level with 30% low roughage; C4_R30: 4% camelina oil level with 30% low roughage; C0_R50: control with 50% medium roughage; C1_R50: M1: 1% camelina oil level with 50% medium roughage; C4_R50: 4% camelina oil level with 50% medium roughage.

The relative abundance of different bacterial phyla observed in our study is presented in Figure 3. Results revealed five major bacterial phyla, namely, *Bacteroidetes* (41.77–51.08%), *Firmicutes* (30.44–43.26%), *Proteobacteria* (4.10–8.95%), *Spirochaetes* (1.33–4.09%), and *Kiritimatiellaeota* (1.14–2.60%), which were observed in all three rations. Moreover, two unique phyla *Fibrobacteres* and *Lentisphaerae* were only detected in the R70:C30 diet. The phylum *Synergistetes* was observed in the medium-roughage (R50:C50) diet only. Three bacterial phyla (*Bacteroidetes, Firmicutes*, and *Proteobacteria*) constituted >98% of total rumen bacteriome. The type of ration and level of CO significantly (*P* < 0.05) affected the bacterial populations. The population of *Firmicutes* (*P* < 0.04) and *Proteobacteria* (*P* < 0.007) was affected by the low and medium level of roughage in all treatment groups. However, supplementation of 2% CO increased (*P* < 0.01) the relative abundance of *Bacteroidetes* in the low-roughage ration. Moreover, the relative abundance of *Spirochaetes* was decreased with the increase in CO level in the high-roughage ration while no difference (*P* > 0.18) was observed in the low- and medium-roughage rations at all CO levels.

#### The Relative Abundance of Bacteria at the Genus Level

**Figure 4:**
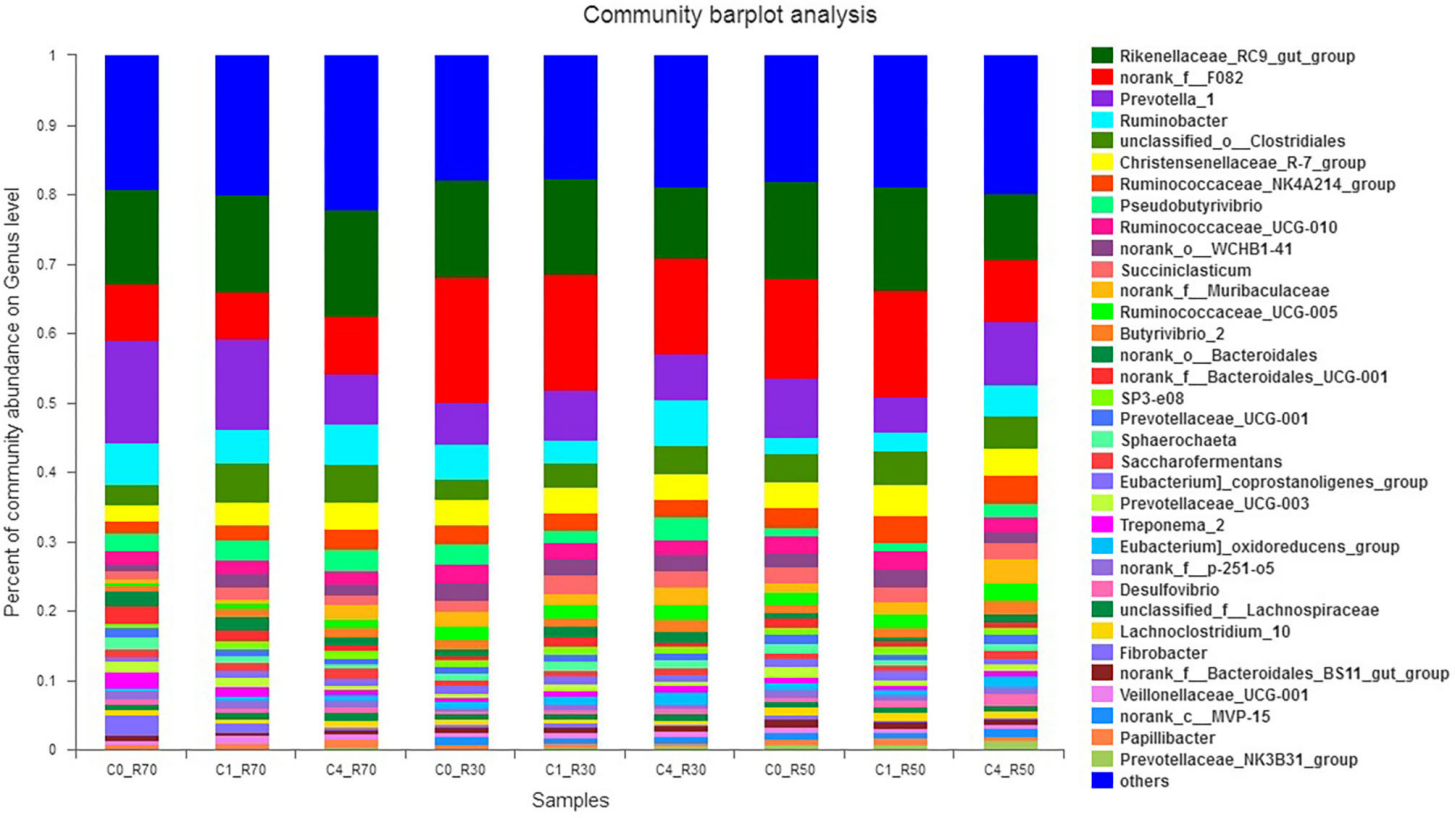
Relative abundance of bacterial genera across different treatment groups. C0_R70: control with 70% high roughage; C1_R70: 1% camelina oil level with 70% high roughage; C4_R70: 4% camelina oil level with 70% high roughage; C0_R30: control with 30% low roughage; C1_R30: M1: 1% camelina oil level with 30% low roughage; C4_R30: 4% camelina oil level with 30% low roughage; C0_R50: control with 50% medium roughage; C1_R50: M1: 1% camelina oil level with 50% medium roughage; C4_R50: 4% camelina oil level with 50% medium roughage.

The effects of ration and CO on the relative abundance of bacterial genera are presented in Figure 4. We detected a total of 364 bacterial genera among which 11 major genera included *Rikenellaceae_RC9_gut_group* (9.5–15.25%), *norank_f_F08, Prevotella_1* (6.82–18.18%), *Ruminobacter* (2.32–6.59%), *unclassified_o_Clostridales* (2.91–5.40%), *Christensenellaceae_R-7_group* (2.41–4.34%), *Ruminococcaceae_NK4A214_group* (1.68–3.93%), *Pseudobutyrivibrio* (1.21–3.06%), *Ruminococcaceae_UCG-010* (1.71–2.76%), *norank_o_WCHB1-41* (1.14–2.57%), and *Succiniclasticum* (1.10–2.75%). These bacterial phyla constituted about 55% of the total bacteriome. The higher level of CO decreased (*P* > 0.109) the abundance of *Rikenellaceae_RC9_gut_group* in low- (R30) and medium- (R50) roughage diets. However, norank_f_F08 was decreased (*P* < 0.007) in high (R70) roughage than other diets. The abundance of the *Prevotella_1* genus was affected (*P* > 0.135) by both the type of ration and treatment. However, a higher CO level increased (*P* < 0.013) the relative abundance of *Ruminobacter* in low- (R30) and medium- (R50) roughage diets than low CO and control groups, but this genus showed similar abundance in high-roughage (R70) groups. Moreover, the abundance of *unclassified_o_Clostridales* was linearly increased (*P* = 0.106) in all three TMRs with the increase in CO as compared to the control. The *Ruminococcus* as cellulolytic bacteria (*Ruminococcaceae_NK4A214_group*) showed variation among different rations, and the highest (*P* < 0.007) abundance was observed in treated medium- (R50) roughage ration. However, *Ruminococcaceae_UCG-005* (ranged from 0.50 to 2.50%) showed a shift in high-roughage rations compared to other rations (*P* < 0.004). A low level of CO favored *Succiniclasticum* in the low roughage ration but reduced (*P* > 0.495) its abundance in the high-roughage diet (R70). The *Pseudobutyrivibrio* increased (*P* < 0.013) in low- and medium-roughage rations supplemented with a high CO level (8%), but no difference in its abundance was observed in high-roughage rations (both treated and untreated). However, a high level of CO increased (*P* = 0.055) the relative abundance of *Butyrivibrio 2* (ranged from 0.80 to 2.03%) in all TMRs.

## Discussion

Our hypothesis that supplementation of camelina oil (rich in PUFA) could affect ruminal bacterial community composition leading to subsequent changes in ruminal fermentation and metabolite production was proved in findings observed in this study.

### Fatty Acid Profile of Camelina Oil

The analysis of the fatty acid profile of CO showed that linolenic acid was dominant fatty acid (>30%), and total unsaturated FA represented >83% of the total fatty acid contents. Previously, total unsaturated fatty acid concentrations up to 86.3, 87.6, and 87.5% have been reported in camelina oil in different studies (25, 39, 40), respectively. However, some studies have also reported lower unsaturated fatty acid fractions (especially, oleic, linoleic, and linolenic acid) ranging from 68 to 74% of total fatty acids (19, 20). These variations in oil composition are mainly due to different extraction methods and seed quality owing to different agro-climatic conditions of crop production. Our study observed a little bit lower levels of UFA (83%) in CO as compared to earlier report (87% UFA observed by (25). A high concentration of UFA can act as an alternate hydrogen sink during bio-hydrogenation (41) resulting in a decrease in CH_4_ production in the rumen (42); however, it may also decrease fiber digestibility (42).

### Methane Production

In the present study, we evaluated the effect of increasing levels of CO on *in vitro* fermentation and methane production. Dietary lipids are one of the promising ways to mitigate methane production in ruminants (18). The use of lipids to reduce enteric CH_4_ production is a better strategy as compared to chemical feed additives like monensin and antibiotics. Recently, a study has reported adverse effects of fatty acids, especially PUFA, on methanogenesis in the rumen (18). The anti-methanogenic effects of PUFA generally get intensified with the increase in double bond number per FA, as suggested by Czerkawski and Clapperton (43). These findings support our observation in the present *in vitro* study. Moreover, another study has shown more severe toxic effects of linolenic acid (C18:3) than linoleic acid (C18:3) contents of linseed on cellulolytic bacteria (44). Our findings on CH_4_ production are consistent with this observation because linolenic acid contents were more than 30% of the total FA in CO used in our study. The decrease in CH_4_ production was observed after 48 h of incubation in response to the supplementation of CO in all rations. Previously, Wang et al. (18) reported that camelina seeds decreased CH_4_ (ml/g DM) in a ration having a roughage-to-concentrate ratio of 60:40.

Our findings are in agreement with Bayat et al. (17), who reported a decrease in daily CH_4_ emission in lactating cows fed with different forage to concentrate ratios (low and high) supplemented with sunflower oil. Moreover, dietary supplementation of CO (at 10 g/kg DM) in a 50% roughage ration has shown to reduce CH_4_ production up to 4.8%. Variable effects of unsaturated fatty acids on CH_4_ emission might be associated with the nature of FA (double bond number per FA), type of oil (free oil or whole seed), and composition (roughage-to-concentrate ratio) of the rations (25).

### Rumen Kinetics

It is the first report on the potential effects of CO on *in vitro* fermentation kinetics using a rumen inoculum from water buffalo. Therefore, due to the scarcity of available information on the subject matter, we compared our findings with studies that used different parts of camelina seed (whole seeds or seed meal) or other vegetable oils. Rumen kinetics such as NH_3_ and VFAs are major end products of rumen fermentation (45). As mentioned before, the majority of earlier studies have used camelina seeds (14, 20) or camelina meal or cake (23, 24), but only two reports are available regarding the use of camelina oil in dairy cattle (25) and fattening sheep (15). All levels of camelina oil linearly decreased rumen pH in R30:C70 ration in the present study; however, no effect of CO on pH was observed in other diets as reported previously (25). Moreover, NH_3_-N was affected in all three diets and linearly decreased with a high concentrate ration. A similar pattern was observed regarding the concentration of MCP. However, Wang et al. (18) found that NH_3_ was high after 24 h of incubation in 60% forage and 40% concentrate diet supplemented with camelina seeds. They suggested that the increase in NH_3_ contents in rumen might indicate accelerated lysis of microbes or reduced nitrogen utilization. However, these findings are in disagreement with the present study, where NH_3_ decreased in high-roughage diet, and this variation might be attributed to the diet composition (different R:C ratios), form (oil vs. seeds), and level of supplemental camelina. Another possible reason for the decrease in NH_3_-N might be the inhibition of bacteria caused by antioxidant compounds of CO (46). Different essential oils and/or plant extracts have also shown to inhibit NH_3_-producing bacteria (*Prevotella spp*. and *R. amylophilus*) up to 77% in sheep (47).

The VFAs are end-products of ruminal fermentation and provide ruminants with about 70% of metabolizable energy (48). The supplementation of CO increased propionate contents in R70:C30 while decreasing it in the other two diets except in the R50:C50 ration (only at 2% CO), which showed a higher propionate than the control group. Additionally, acetate production showed a similar trend like propionate, exhibiting the diet-specific effect of CO on total and individual VFA contents (49). Our findings are in agreement with previous study reporting an increase in the propionate and decrease in total VFA and acetate concentrations in a response to the supplementation of camelina seed (5%) in the diet (55% alfalfa hay: 45% concentrate) under *in vitro* conditions (50). In contrast, Hurtaud and Peyraud (20) observed a significant (*P* < 0.001) decrease in rumen acetate and increase (*P* = 0.014) in propionate in cows fed camelina seeds in the diet (59:41 R:C ratio). Our findings indicate that relatively higher amounts of fiber in the diet and level of CO supplementation can influence molar VFA proportions as well as fiber-degrading bacteria that are particularly sensitive to high-oil diets (44). Therefore, a decrease in acetate and an increase in MCP concentrations observed in R50:C50 and R30:C70 rations suggest a potential inhibition of fiber-degrading bacteria by CO (51).

### Rumen Microbial Population

Our study revealed significant effects of supplementation of camelina oil on ruminal microbial populations, as shown in Table 6. Inhibition of specific microbial population observed in this study might be due to the direct toxic effects of CO on rumen microbes and/or higher non-fermentable fatty acids by replacing readily fermentable carbohydrates (52, 53).

The diet composition significantly affects the diversity and abundance of microbial populations owing to different nutrient contents and fermentation profiles (54). Similar findings have been reported earlier in cows fed high-roughage or high-concentrate diets (55). A significant decrease in methanogenic archaea was observed in goats when diet changed from alfalfa hay to a mixture of oats and alfalfa hay (56). In our study, camelina oil significantly affected the composition of ruminal microbiota, which is mainly attributed to the potential effects of its fatty acid contents. The significant decrease in the number of protozoa and methanogens well-correlated with the results of the CH_4_ yield observed in this study. The rumen archaea use hydrogen to yield CH_4_ to facilitate fiber digestion (57). The inhibition of methanogens is potentially beneficial owing to its significant association with methane emission and animal productivity (57).

The increasing levels of CO levels in diets had no effect on ruminal protozoa (58). However, the decrease in bacterial nitrogen and the number of cellulolytic bacteria have been observed in diets supplemented with 8% dietary lipids (50). Moreover, Bayat et al. (25) reported that CO exhibited no effect on protozoa, total bacteria, methanogens, fungi, and fiber-degrading bacteria. Variable results reported by different studies may be attributed to the dietary form of camelina used in the diet, composition of feed ingredients, roughage-to-concentrate ratio, and dose of oil supplements (25, 49, 59). Another explanation of our findings is the manipulation of rumen biohydrogenation as PUFA (such as linolenic acid) contents of CO could use hydrogen produced by rumen microbes serving as an alternate hydrogen sink (9).

### Rumen Bacterial Diversity

Our study revealed significant effects of CO and ration (roughage-to-concentrate ratio) on ruminal bacterial diversity. Both dietary and treatment factors altered ruminal bacterial communities as well as their richness and diversity indices, as reported previously (13). In addition, shifts in ruminal microbial community and decrease in richness and diversity indices in response to supplementation of camelina seeds as compared to the dietary fat have been reported earlier (13). The study suggested two possible reasons for these findings: (1) higher saturated fatty acid contents of dietary fat (Megalac) posed deleterious effects on rumen environment and (2) toxic effects of PUFA on rumen bacteria such as cellulolytic bacteria.

Our study revealed higher bacterial species richness both in high-roughage diets and in the lower level of CO as revealed by rarefaction curves. The four phyla, including *Bacteroidetes, Firmicutes, Proteobacteria*, and *Spirochaetes*, were detected as major phyla in our study, which is in agreement with earlier reports (13, 60). However, change in roughage-to-concentrate ratio favored two phyla *Fibrobacteres* and *Lentisphaerae* in the R70:C30 diet and one phylum *Synergistetes* in R50:C50 diet.

Two dominant phyla *Firmicutes* and *Bacteroidetes* constitute a major portion of rumen bacteriome. The *Firmicutes* produce propionate and butyrate, while *Bacteroidetes* produce short-chain fatty acids (SCFAs) by fermenting polysaccharides and cellulosic plant material with potential beneficial effects (61–63). In our study, an increase in *Firmicutes* to *Bacteroidetes* ratio was observed in all roughage rations more specifically at higher levels (8%) of CO. Earlier study has reported the potential association of this ratio of two major bacterial phyla (*Firmicutes*: *Bacteroidetes*) with energy homeostasis in animals (adipogenesis) and milk-fat yield in dairy cattle (64). An increase in this ratio in response to CO supplementation in our study correlated well with higher concentrations of propionate and butyrate observed in the present study. Moreover, acetate is produced by *Bacteroidetes* as a result of the fermentation of indigestible carbohydrates, which decreased in all treated TMRs. Camelina oil possesses abundant polyphenols and PUFA (mainly C18:3) contents that exhibited more obvious results against Gram -ve bacteria (13, 18, 44).

At the genus level, no data is available on the effects of the supplementation of CO on the rumen microbiome. Only one study has reported the effects of camelina seeds on microbial diversity (13). Our study revealed shifts in the relative abundance of the *Rikenellaceae RC9 gut group* with a high level of CO in low- and medium-roughage rations, which is in agreement with previous findings observed with the use of Tucumã oil in TMR (65). *Prevotella* was observed as a second major bacterial genus in our present study (Figure 4). Higher and lower levels of CO affected the relative abundance of *Prevotella* spp. that fluctuated among three rations. A low level of CO in the high-roughage ration (R70) showed a higher abundance of *Prevotella* spp. as compared to low- (R30:C70) and medium-roughage (R50:C50) rations. Contrarily, an increase in the relative abundance of *Prevotella* species (*P*. *bryantii* and *P*. *ruminicola*) in response to supplementation of higher levels of plant essential oils has been reported earlier (66). Moreover, dietary protein contents have shown to positively affect the relative abundance of *Prevotella* (60). However, the *Prevotella* abundance was decreased by 8% dietary ether extract, which is in agreement with our findings observed with a high level of CO in the present study (13). Variable findings observed in previous reports might be due to the type of oil, its levels, and the composition of diets. Moreover, studies have also revealed the supportive role of *Prevotella* in fiber digestion and utilization through facilitating other rumen microbes (13, 60, 66, 67). In our study, a decrease in acetate production with a high-roughage ration was observed in response to the shifting of *Ruminococcaceae_UCG-005* in high-roughage rations (60) possibly due to the toxic effects of linolenic acid contents (of camelina oil) on *Ruminococci* spp. (13).

Ruminal fermentation of starch by *Ruminobacter* and *Succiniclasticum* usually yields acetic and succinic acids (68). Our findings regarding the decrease of *Succiniclasticum* in the high-roughage ration are consistent with earlier report (60). On the other side, *Ruminobacter* abundance was decreased by a lower level of CO (2%) in low- and medium-roughage rations but increased with a high level of CO (8%) in all rations. Fermentation of polysaccharides by rumen bacteria (*Butyrivibrio* and *Pseudobutyrivibrio*) produces formic, butyric, and acetate acids in the rumen (69). The present study showed a higher level of butyric acid with supplementation of CO at 4 and 6% levels in all rations, which is mainly attributed to the increased number of *Butyrivibrio* species. Additionally, a decrease in butyric acid contents with the increase in roughage level was observed; however, the relative abundance of *Pseudobutyrivibrio* and *Butyrivibrio* showed a similar trend in all three TMRs and decreased with supplementation of CO at a lower level for low- and medium-roughage diets. However, Dai et al. (13) reported that supplementation of camelina seeds decreased (*P* < 0.05) *Butyrivibrio* without affecting the relative abundance of *Pseudobutyrivibrio*. A possible reason for decreased *Butyrivibrio* and *Pseudobutyrivibrio* with the low CO level and their increase with the high CO level might be attributed to the first shock of the toxic effects of linolenic acid on cellulolytic bacteria. Moreover, *Pseudobutyrivibrio* is more resistant than *Butyrivibrio* in the presence of C18:2n6, and C18:3n3, as well as *Butyrivibrio*, has significant negative association and sensitivity to toxic effects of PUFA (47, 70). However, previous study has reported a linear decrease in *Butyrivibrio* and *Pseudobutyrivibrio* with increasing levels of starch while the linear increase was observed with increasing fiber contents in the diet, which is consistent with our findings (60). This shows that *Butyrivibrio* and *Pseudobutyrivibrio* preferably ferment structural carbohydrates to yield energy. Additionally, similar functions of *Eubacterium* and *Butyrivibrio* regarding the fermentation of structural carbohydrates have been reported earlier (71). Conclusively, our study revealed desirable effects of camelina oil on rumen microbiota and fermentation kinetics while reducing CH_4_ emission which is advantageous in terms of increasing feed efficiency and reducing environmental hazards.

## Conclusions

Camelina oil reduced CH_4_ production in total mixed rations, revealing its potential as a feed additive to mitigate overall methane emissions from livestock production. Supplementation of CO at the 8% level had adversely affected the diversity and evenness of bacterial populations. However, CO also increased *Firmicutes* to *Bacteroidetes* ratio in all rations particularly at a higher level of supplementation (8%). Camelina oil also negatively affected the *Prevotella* in both low- and medium-roughage diets while increasing the abundance of *Ruminobacter* and *Pseudobutyrivibrio*, revealing its potential to favor the production of formate, butyrate, and acetate. Conclusively, our study revealed that supplementation of 4–6% CO in dairy rations is suitable to improve nutrient digestion and utilization while reducing CH_4_ production. However, further *in vivo* studies are required to confirm our findings and to optimize the best level of CO to improve feed efficiency in ruminants while mitigating methane emanation.

## Data Availability Statement

The datasets presented in this study can be found in online repositories. The names of the repository/repositories and accession number(s) can be found at: https://www.ncbi.nlm.nih.gov/, SRR10072455.

## Ethics Statement

The animal study was reviewed and approved by Ethics Committee of Guangxi Buffalo Research Institute, Chinese Academy of Agriculture Sciences, China.

## Author Contributions

HE: conceptualization, data curation, and writing—original draft. HE, ML, and FH: methodology. FH: software. CY: validation and supervision. HE and FH: formal analysis and investigation. ML, LP, XL, KP, and CY: resources. FH and CY: writing—review & editing. HE and ML: project administration. CY: funding acquisition. All authors contributed to the article and approved the submitted version.

## Conflict of Interest

The authors declare that the research was conducted in the absence of any commercial or financial relationships that could be construed as a potential conflict of interest.
